# The necessity of proactive measures from healthcare providers highlighted by delayed breast cancer diagnosis due to COVID‐19: A case report

**DOI:** 10.1002/ccr3.7919

**Published:** 2023-09-15

**Authors:** Yudai Kaneda, Akihiko Ozaki, Mira Namba, Toyoaki Sawano, Masahiro Wada, Hiroaki Saito, Yoshiaki Kanemoto, Tomohiro Kurokawa, Masaharu Tsubokura, Kazunoshin Tachibana, Tetsuya Tanimoto, Tohru Ohtake, Tomozo Ejiri, Hiroaki Shimmura, Norio Kanzaki

**Affiliations:** ^1^ School of Medicine Hokkaido University Sapporo Japan; ^2^ Department of Breast and Thyroid Surgery Jyoban Hospital of Tokiwa Foundation Iwaki Japan; ^3^ Department of Gastrointestinal Tract Surgery Fukushima Medical University Fukushima Japan; ^4^ School of Medicine Keio University Tokyo Japan; ^5^ Department of Surgery Jyoban Hospital of Tokiwa Foundation Iwaki Japan; ^6^ Utsunomiya Central Clinic Utsunomiya Japan; ^7^ Department of Internal Medicine Soma Central Hospital Soma Japan; ^8^ Department of Medical Epigenomics Research Fukushima Medical University Fukushima Japan; ^9^ Department of Radiation Health Management Fukushima Medical University School of Medicine Fukushima Japan; ^10^ Department of Breast Surgery Fukushima Medical University Fukushima Japan; ^11^ Department of Internal Medicine Jyoban Hospital of Tokiwa Foundation Iwaki Japan; ^12^ Department of Urology Jyoban Hospital of Tokiwa Foundation Iwaki Japan

**Keywords:** breast cancer, COVID‐19, disaster, Japan

## Abstract

**Key Clinical Message:**

During disasters, multiple factors can cause significant delays in medical visits. Regular patient monitoring, high‐risk individual alerts, and telemedicine enhancements can potentially alleviate these issues and ensure timely interventions.

**Abstract:**

During the COVID‐19 pandemic, a Japanese woman in her 70s delayed her regular breast cancer checkup for over 2 years. During disasters, health priorities tend to decline, necessitating proactive measures from healthcare providers, such as augmenting collaboration among healthcare professionals and identifying high‐risk individuals.

## 1 INTRODUCTION

Breast cancer is the most common cancer in women, with an estimated 685,000 fatalities worldwide in 2020.^1^ Fortunately, with the widespread availability of screening and the advancement of medical technology, the survival rate of breast cancer patients in developed countries has exceeded 80%.^2^ Meanwhile, the mortality rate has been reported to increase by up to two to three times with a delay of only 12 weeks in the initial diagnosis and subsequent treatment.^3^ Therefore, providing an appropriate approach for each patient's situation is essential to encourage patient screening and consultation for early detection.^4^

Since the 2011 triple disaster (earthquake, tsunami, and nuclear disaster) in Fukushima, Japan, more attention has been paid to disasters and crises as social factors affecting management of various conditions such as breast cancer screening and consultations. Several reports have indicated that breast cancer patients in Fukushima were reluctant to receive medical consultations after the disaster, resulting in prolonged refrains, with mortality rates increasing among those who delayed their consultations.^5^, ^6^, ^7^ Furthermore, under the ongoing coronavirus disease 2019 (COVID‐19) pandemic, breast cancer patients' refraining from seeing doctors and postponement or cancellation of surgeries and examinations have also been reported.^8^, ^9^

Several factors have been named as relevant to delays in medical visits after disasters and crises, including reduced prioritization of health due to significant changes in the surrounding environment and reduced access to medical care due to crumbling infrastructure.^7^, ^10^, ^11^ In addition, a post‐disaster case study in Fukushima indicated that reduced support from family and friends was associated with delays in medical visits.^12^ Refraining from hospital visits due to the fear of being infected with the COVID‐19 can also be interpreted in this context.^9^, ^13^ Indeed, in Japan, the overall number of hospitalizations and outpatient visits decreased by 27% and 22%, respectively, in May 2020.^14^ This percentage may also have decreased because the guidelines allowed COVID‐19‐positive patients to be treated at home or in designated facilities if they were mildly ill or asymptomatic in certain regions of Japan.^15^ Further, personality factors have been also noted as possible factors associated with delays in hospital visits.^16^, ^17^

However, there have not been enough studies on breast cancer patients who delayed their medical visits that evaluated related factors such as lower health priorities, personality, diminished availability of healthcare services owing to deteriorating infrastructure, and support from the surrounding community in actual cases of disasters and crises. Therefore, we assessed the consultation delay of a breast cancer patient who visited our hospital for suspected breast cancer symptoms and interrupted her visits for as long as 2 years due to the state of emergency against the COVID‐19 pandemic before diagnosed with breast cancer in Fukushima, Japan.

## 2 CASE PRESENTATION

A Japanese woman in her 70s with a previous history of hypertension and no relevant family history visited another hospital in 1999 for left nipple non‐bloody discharge, but a cytological examination of the discharge did not demonstrate any malignant cells. Since then, she has undergone checkups every 6 months without any malignancy being detected.

She noticed that her discharge color was turning reddish about 2 months before and visited our hospital in January 2019. The mammography and ultrasonography demonstrated a well‐defined, smooth‐margined oval mass with calcification, which appeared to be a series of masses from the left E to the AB area. As the assessment of these imaging studies was category 3 and malignancy could not be excluded, we performed a core needle biopsy under the guide of ultrasonography. The biopsy finding was mastopathy and did not show a presence of malignancy, and we judged that there is no discordance between the pathological and imaging findings. However, still considering a possibility of malignancy, we instructed her to revisit our hospital in 6 months.

When she visited our hospital in July 2019, no enlargement of the mass was identified on mammography and ultrasonography. While the bloody discharge persisted, its amount did not show a clinically meaningful increase. Consequently, we did not conduct a core needle biopsy but only performed a cytological examination. The result of cytological examination was class 2, and we instructed the patient to revisit us 1 year later.

Subsequently, the COVID‐19 pandemic had begun in Japan since January 2020, leading to a declaration of a state of emergency in Japan around April 2020. This statement has discouraged Japanese citizens to go out including hospital visits.^14^ Thus, she failed to visit our hospital at the expected time and came to consult in March 2022 with the primary symptom of increased bloody discharges, without lumps or any other signs of noticeable abnormality. On examination, a mass was found just under the left nipple without indrawing of the nipple or ulceration of the skin/exfoliative lesion. Mammography and breast ultrasonography showed a 50‐mm‐sized, well‐defined, smooth‐margined oval mass. Thus, we again conducted a core needle biopsy and invasive ductal carcinoma (estrogen receptor >90%, progesterone receptor >90%, human epidermal growth factor receptor 2 score 0, Ki67 31.2%) was detected. As additional imaging studies such as magnetic resonance imaging, computed tomography, and positron emission tomography did not reveal clinical meaningful lymph swelling or distant metastasis, she was diagnosed with cT2N0M0 Stage IIA breast cancer.

On April 2022, she underwent mastectomy and sentinel lymph node biopsy. A rapid examination revealed no metastasis to the sentinel node, so an axillary dissection was omitted. Based on final pathology, she was diagnosed with pT3 (55 mm) N0M0 Stage IIB, breast invasive ductal carcinoma, nuclear grade 3, and histological grade 3. Given that she had a recurrence score of 13 on the 21‐gene assay (Oncotype DX Recurrence Score, Genomic Health), she was treated only with hormone therapy.

The patient was then interviewed about the background of the delay in visiting our hospital. She explained that she decided to wait to see us after the outbreak of the COVID‐19 pandemic because she had thought that medical facilities would be in a difficult situation, and she believed her disease was already benign. Later, around January 2021, she noticed an increase in the secretion of discharge and an intensification of its redness. Despite this, she opted to wait for the pandemic to subside. However, with the ongoing uncertainty regarding the end of the pandemic and her symptoms progressively worsening, she eventually made the choice to reach out to the hospital in November 2021. Since the increase in secretions was not mentioned at that time, an administrative staff member who answered the phone at the breast cancer outpatient unit did not relay the information to the medical doctor. Consequently, a regular appointment was scheduled for the patient in March 2022. She received the two COVID‐19 vaccine shots in July 2021 and a booster shot in March 2022. Consequently, when she eventually visited the hospital, she did not harbor strong concerns about contracting COVID‐19.

The reason for not rushing to see the doctor was that she had not been diagnosed with breast cancer previously despite multiple investigations. Despite describing herself as an easy‐going and laid‐back individual, her score on the Japanese version of the revised Life Orientation Test (LOT‐R) was 12.^18^ This indicates that she possesses less optimistic attitudes compared to the general population.

Regarding her family, her daughter had already matured and was living away from Iwaki city, but she was living with her husband. However, she also has the tendency to keep everything to herself and did not consult with her husband, daughter, or friends during this period.

## 3 DISCUSSION

We reported a patient who was diagnosed with breast cancer after refraining from seeking a medical consultation for more than 2 years during the COVID‐19 pandemic in Japan. The failure to conduct a follow‐up consultation after a year had passed, coupled with the lack of a proactive system to inquire about symptoms when she called, significantly contributed to the prolonged delay, exposing serious deficiencies in the healthcare system.

Notably, the lack of effective communication regarding crucial information, particularly changes in symptoms, between administrative and medical staff resulted in delayed intervention for this case. Effective coordination in healthcare is crucial for the timely management of breast cancer patients particularly during this unprecedented crisis.^19^ Concurrently, it is imperative to promote timely healthcare by maintaining active communication with patients, while being mindful of the merits and demerits of the rapidly expanded online consultations in the wake of the COVID‐19 pandemic.^19^, ^20^

Moreover, several other factors should be considered to understand why such a lengthy delay occurred. First, in disasters and crises, the importance of personal health tends to decrease temporarily, particularly in diseases such as breast cancer, which progresses relatively slowly.^5^, ^6^, ^7^, ^10^ Moreover, in this case, the failure to diagnose cancer in multiple previous visits may have lowered the priority of breast symptoms in the pandemic. Indeed, previous studies have also shown that a previous benign diagnosis can decrease a patient's suspicion of cancer.^21^

Another important characteristic is that she did not adequately consult with the people around her about her condition. There have been reports of cancer patients who delayed visiting hospitals due to the loss of opportunities to communicate with their surrounding family and friends about their health conditions after the triple disaster.^7^ While this patient did not lose any family members to COVID‐19, her failure to actively seek support from her surroundings may have resulted in a lack of response, leading to a prolonged delay in seeking medical consultation. In this respect, this can be said to be a matter of personality, but it is also a matter of family and surrounding support. Thus, beyond external factors like disasters, it is particularly essential to consider the individual's home environment and personal characteristics in crafting a personalized response.

Of course, there are limitations to what we can state from a single case. It is impossible to determine which factors contributed specifically to the results and to what extent. Nevertheless, one thing that is clear from this case is the possibility of long‐term delays in hospital visits due to the simultaneous involvement of a variety of factors that may contribute to such delays, and it will be necessary for health care professionals to be fully aware of their patients' backgrounds on a daily basis.

Moreover, a coherent strategy is reminders. For example, our hospital routinely requests patients to make their appointments when they revisit us in more than 1 year. Thus, we could not comprehensively identify those who had not made an appointment or seen a doctor 1 year later, especially under the COVID‐19 pandemic, thereby leading to the above situation. To bridge this gap and establish a foundation that prevents interruption of treatment even during disasters, it is crucial to be mindful in normal consultations about how to continuously remind patients by integrating new technologies, such as short message services, even with limited manpower.^22^ Moreover, since the COVID‐19 pandemic began, its impact on access to healthcare and prioritization has underscored the need for a more robust telemedicine and outreach system, particularly for chronic diseases and suspect cases.^23^ Lastly, it is important to listen to the patient's personality and family environment during regular medical consultations and through collaboration with other professionals, such as medical social workers, to guide the patient to a timely visit to the hospital, even in times of crisis.

In conclusion, we experienced a Japanese woman in her 70s who delayed her regular breast cancer checkup for over 2 years, resulting in a late‐stage diagnosis. The delay in the patient's visit, influenced by factors such as reduced health prioritization, personality, infrastructure disruptions, and lack of surrounding support, revealed the hospital's inadequate approach, highlighting a significant challenge during disasters. Hence, it is vital to enhance hospital functions through the introduction of telemedicine, the strengthening of collaboration among healthcare workers, and proactive identification of high‐risk individuals during disasters, in addition to carefully monitoring patients' conditions on a regular basis.

## AUTHOR CONTRIBUTIONS

**Yudai Kaneda:** Conceptualization; writing – original draft. **Akihiko Ozaki:** Conceptualization; writing – original draft. **Mira Namba:** Writing – review and editing. **Toyoaki Sawano:** Writing – review and editing. **Masahiro Wada:** Writing – review and editing. **Hiroaki Saito:** Writing – review and editing. **Yoshiaki Kanemoto:** Writing – review and editing. **Tomohiro Kurokawa:** Writing – review and editing. **Masaharu Tsubokura:** Writing – review and editing. **Kazunoshin Tachibana:** Writing – review and editing. **Tetsuya Tanimoto:** Writing – review and editing. **Tohru Ohtake:** Supervision. **Tomozo Ejiri:** Supervision. **Hiroaki Shimmura:** Supervision. **Norio Kanzaki:** Supervision.

## FUNDING INFORMATION

Not applicable.

## CONFLICT OF INTEREST STATEMENT

Akihiko Ozaki receives personal fees from MNES Inc and Kyowa Kirin, outside the submitted work. Also, Tetsuya Tanimoto receives personal fees from MNES Inc, and Bionics co., ltd. outside the submitted work. No other authors reported conflicts of interest.

## CONSENT

Written informed consent was obtained from the patient for publication of this case report and any accompanying images. A copy of the written consent is available for review by the Editor‐in‐Chief of this journal.

## Data Availability

Data sharing is not applicable to this article as no new data were created or analyzed in this study.
